# Secondary Circulating Prostate Cells Predict Biochemical Failure in Prostate Cancer Patients after Radical Prostatectomy and without Evidence of Disease

**DOI:** 10.1155/2013/762064

**Published:** 2013-03-31

**Authors:** Nigel P. Murray, Eduardo Reyes, Nelson Orellana, Cynthia Fuentealba, Leonardo Bádinez, Ruben Olivares, José Porcell, Ricardo Dueñas

**Affiliations:** ^1^Division of Medicine, Hospital de Carabineros de Chile, Simón Bolívar 2200, Ñuñoa, 7770199 Santiago, Chile; ^2^Instituto de Bio-Oncología, Avenida Salvador 95, Oficina 95, Providencia, 7500710 Santiago, Chile; ^3^Circulating Tumor Cell Unit, Faculty of Medicine, Universidad Mayor, Renato Sánchez 4369, Las Condes, 7550224 Santiago, Chile; ^4^Faculty of Medicine, Universidad Diego Portales, Manuel Rodriguez Sur 415, 8370179 Santiago, Chile; ^5^Urology Division, Hospital de Carabineros de Chile, Simón Bolívar 2200, Ñuñoa, 7770199 Santiago, Chile; ^6^Foundation Arturo Lopez Perez, Rancagua 899, Providencia, 7500921 Santiago, Chile; ^7^Division of Medicine, Hospital de Carabineros de Chile, Simón Bolívar 2200, Ñuñoa, 7770199 Santiago, Chile

## Abstract

*Introduction*. Although 90% of prostate cancer is considered to be localized, 20%–30% of patients will experience biochemical failure (BF), defined as serum PSA >0.2 ng/mL, after radical prostatectomy (RP). The presence of circulating prostate cells (CPCs) in men without evidence of BF may be useful to predict patients at risk for BF. We describe the frequency of CPCs detected after RP, relation with clinicopathological parameters, and association with biochemical failure. *Methods and Patients*. Serial blood samples were taken during followup after RP, mononuclear cells were obtained by differential gel centrifugation, and CPCs identified using standard immunocytochemistry using anti-PSA monoclonal antibodies. Age, pathological stage (organ confined, nonorgan confined), pathological grade, margin status (positive, negative), extracapsular extension, perineural, vascular, and lymphatic infiltration (positive, negative) were compared with the presence/absence of CPCs and with and without biochemical failure. Kaplan Meier methods were used to compare the unadjusted biochemical failure free survival of patients with and without CPCs. *Results*. 114 men participated, and secondary CPCs were detected more frequently in patients with positive margins, extracapsular extension, and vascular and lymphatic infiltration and were associated with biochemical failure independent of these clinicopathological variables, and with a shorter time to BF. *Conclusions*. Secondary CPCs are an independent risk factor associated with increased BF in men with a PSA <0.2 ng/mL after radical prostatectomy, but do not determine if the recurrence is due to local or systemic disease. These results warrant larger studies to confirm the findings.

## 1. Introduction

In the PSA era more than 90% of prostate cancer cases are considered to be localized at the time of diagnosis; however, 20%–30% of these patients will experience biochemical failure, usually in the first two years after surgery [1, 2]. Biochemical failure may occur as late as 10 to 15 years after primary treatment, with a mean time of 8 years from biochemical failure to the appearance of clinical metastasis [1]. This suggests the persistence of tumor cells in a state of either complete or near dormancy prior to metastatic progression. It has been reported that disseminated tumor cells in bone marrow predict biochemical failure after radical prostatectomy [3]. Disseminated tumor cells (DTCs) in bone marrow aspirates were detected in 57% of patients without evidence of disease after radical prostatectomy and detected in 45% of patients 5 years after surgery. These patients positive for DTCs in bone marrow aspirates had a nearly 7-fold increased risk of biochemical failure compared with patients DTC negative [3]. Although the risks of complications after bone marrow aspiration are estimated to be approximately 0.4% [4], the use of circulating prostate cells in blood would be easier to implement. It has been recently published that circulating prostate cells (CPCs) are phenotypically identical to DTCs and that both represent circulating cells in two different tissue compartments in patients with prostate cancer and not true micrometastasis [5]. Early dissemination of cancer cells regardless of stage, grade, or tumor volume has been previously reported; dissemination first occurs to the neurovascular structures and then onto the circulation [6]. The majority of these cells will be eliminated by host defense mechanisms; however a small number will implant in distant tissues, survive, and in time proliferate. These cells will not be eradicated by radical prostatectomy and may later be detected in the circulation, secondary CPCs.

The purpose of this study was to describe the prevalence of secondary CPCs after radical prostatectomy and determine if this information would be clinically relevant and if there was an association with biochemical failure.

## 2. Methods and Patients

### 2.1. Patient Selection

From January 2009 to December 2011 blood samples from consecutive prostate cancer patients were prospectively collected for the purpose of detecting CPCs and evaluating whether these cells were correlated with clinical outcomes. All patients who had undergone radical prostatectomy at the author's institution and all those seen during followup were invited to participate. Samples were taken from men at least three months after surgery and considered to be without evidence of disease. This was defined as being bone scan negative and a serum PSA <0.20 ng/mL. A group of men with a serum PSA of 0.2–1.0 ng/mL and bone scan negative was selected to represent men with biochemical failure. All samples were obtained after written informed consent and collected using protocols approved by the local ethics committee.

### 2.2. Sample Collection and Cell Enrichment

8 mL of venous blood was collected in tubes containing EDTA (Beckson-Vacutainer). Mononuclear cells were obtained using gel differential centrifugation using Histopaque 1,077 (Sigma-Aldrich) at room temperature according to manufacturer's instructions and finally washed 3 times in phosphate-buffered saline pH 7.4 (PBS). The pellet was resuspended in 100 *μ*L of autologous plasma and 25 *μ*L used to prepare each slide (silanized DAKO, USA). The slides were air-dried for 24 hours and finally fixed in a solution of 70% ethanol, 5% formaldehyde, and 25% PBS for 5 minutes and then washed 3 times with PBS.

### 2.3. Identification of CPCs

Slides were processed within 1 hour of fixation and incubated with anti-PSA clone 28A4 (Novocastra Laboratory, UK) in a concentration of 2.5 *μ*g/mL for 1 hour at room temperature and identified using a detection system based on alkaline phosphatase-antialkaline phosphatase (LSAB2 DAKO, USA) with new fuchsin as the chromogen. To permit the rapid identification of positive cells there was no counterstaining with Mayer's hematoxylin. Levamisole (DAKO, USA) was used as an inhibitor of endogenous alkaline phosphatase. Positive and negative controls were processed in the same way.

**Figure 1:**
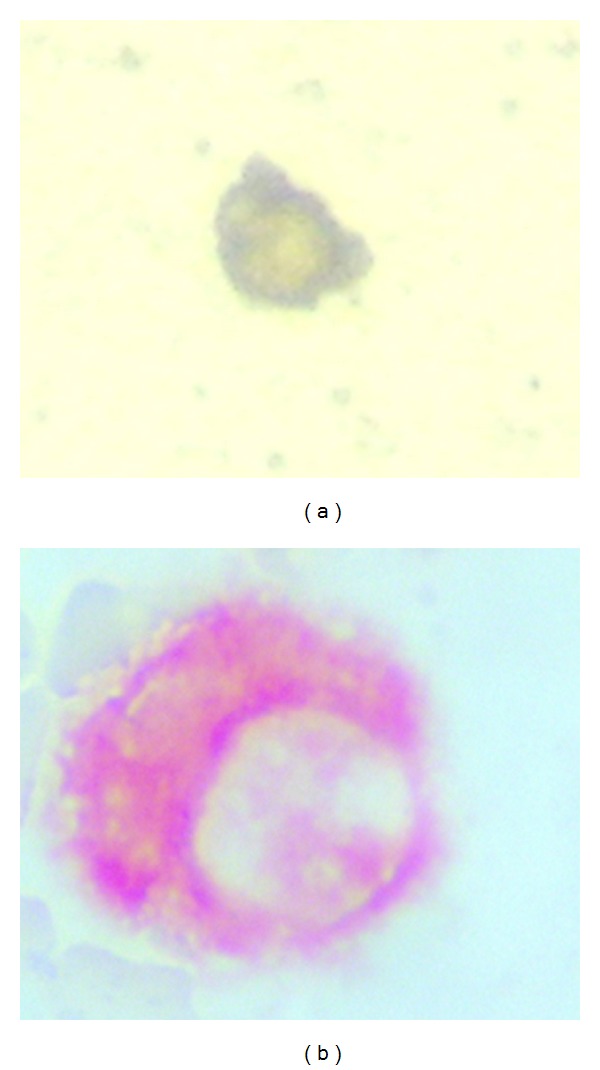
(a) Leucocyte (PSA negative). (b) CPC PSA positive.

Definition of secondary CPCs using the criteria of ISHAGE was used to identify immunostained cells (7), a cell positive for PSA with a nucleus (Figures 1(a) and 1(b)). Samples were analyzed at low power and photographed at a magnification of 400x using a digital camera, Samsung Digimax D73, and processed with the Digimax program for Windows 98. The immunocytochemical evaluation was performed by a single person, blinded to the clinical details using a coded system.

### 2.4. Statistical Methods

Descriptive statistics were used to compare demographic and disease characteristics of patients with and without biochemical failure. Univariate comparisons were tested using chi-squared and Kaplan Meier methods were used to compare the unadjusted free from biochemical failure of patients with and without CPCs detected. Age, pathological stage (organ confined, nonorgan confined), pathological grade, margin status (positive, negative), extracapsular extension (positive, negative), and perineural, vascular, and lymphatic infiltration (positive, negative) were compared with the presence/absence of CPCs and with and without biochemical failure.

Because the time between radical prostatectomy and the blood sampling was not standardized, two separate models were considered. In the first model, the time under observation started at the date of radical prostatectomy. In the second the time under observation started at the time of blood sampling after surgery. Patients who did not experience biochemical failure were censored at the date of last followup.

## 3. Results

114 men with a mean age of 71.5 ± SD 8.2 years participated.  Table 1 shows the distribution of patients according to PSA levels, pathological stage at diagnosis, and median time from surgery to blood sampling. Men in Group 1 had significantly less pT3 disease (*P* = 0,04 chi-squared) than Group 2.

Secondary CPCs were detected in 10/28 (35.7%) men in Group 1, 27/64 (42.2%) in Group 2, and 15/22 (68.2%) in Group 3. There was a significant tendency of increased frequency of CPC detection with increasing serum PSA (*P* = 0.002 chi-squared for tendency) with a relative risk of 1.00, 1.31, and 3.86, respectively.

Secondary CPCs were detected more frequently in patients with positive margins, extracapsular extension, and vascular and lymphatic infiltration but not with perineural infiltration (Table 2). There was a trend with increasing frequency of CPC detection with pathological stage (*P* = 0.002 chi-squared for trends) with a relative risk of 1.00, 3.63 and 10.83 for stages pT1, pT2, and pT3, respectively, and with increasing Gleason score (*P* = 0.015) with a relative risk of 1.00, 7.41, 4.00, and 44.00 for Gleason 4, 5 + 6, 7 and 8 + 9, respectively.

### 3.1. Analysis of Biochemical Failure in Groups 1 and 2

7/28 (25.0%) of men in Group 1 and 23/64 (35.9%) of men in Group 2 experienced biochemical failure within the study period (*P* = 0.37 chi-squared). Comparing men with and without biochemical failure, there were no significant differences in the number of patients with margins positive 9/22 versus 22/70 (*P* = 0.64 chi-squared) or capsule compromised 14/38 versus 16/54 (*P* = 0.53 chi-squared). Biochemical failure was more frequent in men with perineural infiltration 25/55 versus 5/37 (*P* = 0.001 chi-squared) and vascular infiltration 10/18 versus 20/74 (*P* = 0.021 chi-squared) but not with lymphatic infiltration 8/16 versus 22/77 (*P* = 0.08 chi-squared).

There was a trend of increasing biochemical failure with increasing Gleason score, comparing Gleason 4, Gleason 5 + 6, Gleason 7, and Gleason 8 + 9, (*P* = 0.05 chi-squared for trends), with a relative risk of 1.00, 6.00, 8.70, and 9.60, respectively (Table 3).

### 3.2. Association of CPC Status and Clinicopathological Parameters with Biochemical Failure

Incorporating the detection of CPCs with the pathological parameters showed different results. 25/38 (65.8%) men CPC positive experienced biochemical failure in comparison with 5/56 (8.9%) of men CPC negative (*P* = 0.0001 chi-squared).

#### 3.2.1. CPC and Margin Status (Table 4(a))

Men CPC (+) margin (+) were more likely to experience biochemical failure than men CPC (−) margin (+), 9/15 versus 0/7 (*P* = 0.022 chi-squared); likewise men CPC (+) margin (−) were more likely to experience biochemical failure than men CPC (−) margin (−), 17/22 versus 4/50 (*P* = 0.0001 Chi-squared) (Table 6). Comparing CPC (+) margin (+) with CPC (+) margin (−) there was no significant difference (*P* = 0.16 chi-squared); similarly there was no difference between CPC (−) margin (+) and CPC (−) margin (−) (*P* = 1.00 Fisher two-tailed).

#### 3.2.2. CPC and Extracapsular Extension (Table 4(b))

Men CPC (+) capsule (+) were more likely to experience biochemical failure than men CPC (−) capsule (+), 13/33 versus 1/16 (*P* = 0.0008 Fisher two-tailed); likewise men CPC (+) capsule (−) were more likely to experience biochemical failure than men CPC (−) capsule (−) (*P* = 0.0001, Fisher two-tailed). Comparing CPC (+) capsule (+) with CPC (+) margin (−) there was no significant difference (*P* = 0.47); equally there was no significant difference between CPC (−) capsule (+) with CPC (−) capsule (−) (*P* = 1.00 Fisher two-tailed).

#### 3.2.3. CPC and Perineural (PN) Infiltration (Table 4(c))

Men CPC (+) PN (+) were more likely to experience biochemical failure compared with CPC (−) PN (+) 20/25 versus 5/30 (*P* = 0.0001 chi-squared), similarly for men CPC (+) PN (−) versus CPC (−) PN (−), 5/11 versus 0/26 (*P* = 0.001, Fisher two-tailed). Comparing men CPC (+) PN (+) versus CPC (+) PN (−) there was no significant difference (*P* = 0.056 Fisher two-tailed). Similarly for CPC (−) PN (+) versus CPC (−) PN (−) there was no significant difference (*P* = 0.055, Fisher two-tailed).

#### 3.2.4. CPC and Vascular (V) Infiltration (Table 4(d))

There was no significant difference in biochemical failure between CPC (+) V (+) and CPC (−) V (+) 10/16 versus 0/2 (*P* = 0.18 Fisher two-tailed); however, men CPC (+) V (−) were more likely to experience biochemical failure than men CPC (−) V (−) 15/18 versus 5/56 (*P* = 0.0001 Fisher two-tailed). Comparing V (+) versus V (−) in men CPC (+) there was no difference (*P* = 0.25 Fisher two-tailed) or comparing V (+) versus V (−) in men CPC (−) (*P* = 1.00 Fisher two-tailed).

#### 3.2.5. CPC and Lymphatic (L) Infiltration (Table 4(e))

There was no significant difference in biochemical failure between CPC (+) L (+) and CPC (−) L (+) (*P* = 0.2 Fisher two-tailed); however men CPC (+) L (−) were more likely to experience biochemical failure than men CPC (−) L (−) (*P* = 0.0005 Fisher two-tailed). There were no significant differences in biochemical failure between CPC (+) L (+) versus L (−) or CPC (−) L (+) versus L (−).

#### 3.2.6. CPC and Gleason Score (Table 4(f))

There was no significant difference in biochemical failure in relation to the Gleason score in men CPC (+) or in relation to the Gleason score in men CPC (−) nor was there a trend for increasing failure with increasing Gleason score in the two groups, CPC (+) and CPC (−).

#### 3.2.7. Frequency of Biochemical Failure in CPC Positive and Negative Men with Time from Surgery

**Figure 2:**
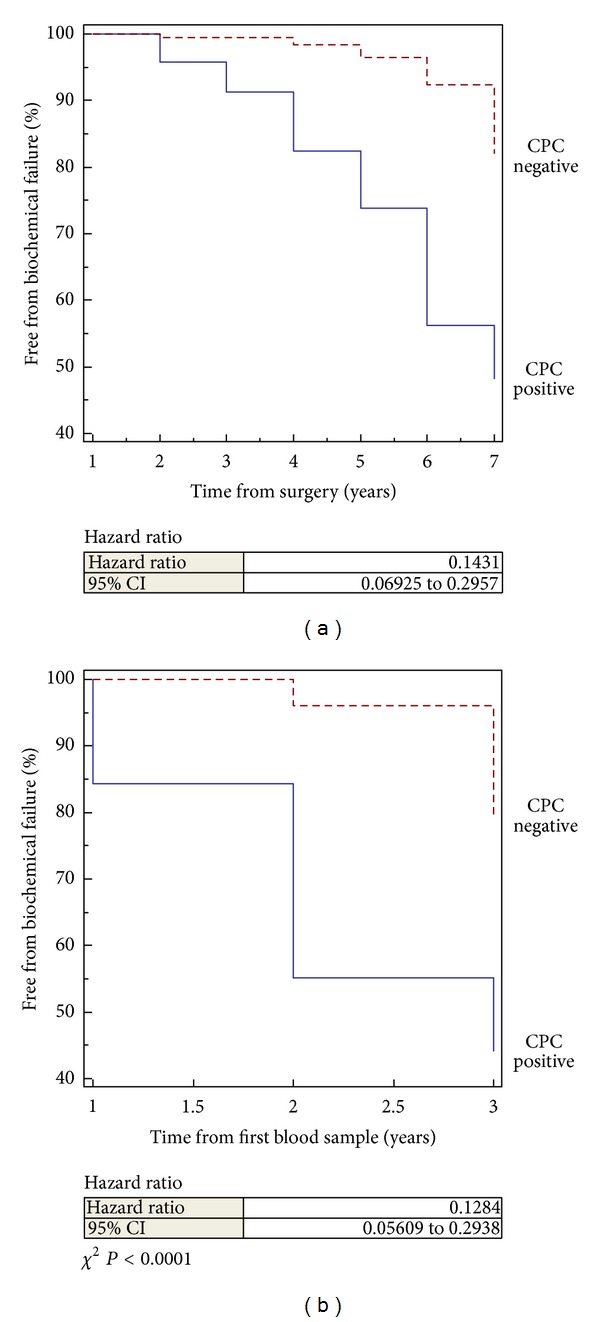
Kaplan-Meier plot time free of biochemical failure from time of (a) surgery and (b) blood sampling.

Men CPC positive had a higher frequency of biochemical failure during the first 5 years after surgery; however both CPC positive and negative men continued to experience biochemical failure after 5 years (Table 5 and Figure 2).

#### 3.2.8. Frequency of Biochemical Failure in CPC Positive and Negative Men with Time from First Blood Sample

Men CPC positive had a higher frequency of biochemical failure at 1, 2, and 3 years of followup (Figure 2).

## 4. Discussion

The object of this study was to describe the prevalence of CPCs after radical prostatectomy. The high rate of dissemination prior to treatment has been used as a sequential method to detect prostate cancer [7]; however with surgical removal of the primary tumor, the primary source of circulating tumor cells is eradicated. Circulating tumor cells detected after primary treatment (secondary CPCs) therefore disseminate from a micrometastatic focus which may be local from the prostate bed or surrounding tumor or systemic from distant tissues. 40.2% of cases without evidence of biochemical failure had secondary CPCs detected using standard gel differential centrifugation and immunocytochemistry, including patients initially CPC negative and with the appearance of secondary CPCs >5 years after surgery. The 40.2% of men positive for secondary CPCs is less than the 57% of men with DTCs after prostatectomy reported by Morgan et al. [3]. However, our study group included stage T1 patients which may explain this difference. An alternative explanation is that CPCs are actively disseminating tumor cells; thus in patients without active dissemination of tumor cells but with dormant bone marrow micrometastasis the frequency of CPC detection will be less as has been suggested [5].

The population studied experienced biochemical failure in 32.6% of patients, comparable to the internationally published data. Known clinical-pathological risk factors correlated with the occurrence of biochemical failure were associated with a higher frequency of CPC detection, except for perineural invasion. 

Not all these clinic-pathological risk factors were associated with biochemical failure; positive margins and compromise of the capsule by tumor were not associated. Perhaps more importantly the presence of secondary CPCs was associated with biochemical failure independent of these clinicopathological variables. Assuming that patients negative for CPC detection and known risk factor had the least possibility of biochemical failure, the relative risk of failure was significantly higher in patients CPC positive independent of the status of the clinical variable.

Surprisingly the presence of positive margins was not associated with biochemical failure, maybe due to the short time of followup in our patients. Ploussard et al. [8] reported that although positive margins were detected in 25.6% of cases, only 14.7% of the 1943 patients studied experience biochemical failure, the 5-year biochemical free survival being reported as 57.5% in margin positive patients compared with 84.4% in men margin negative. In men with pT2N0 cancer positive margins were not associated with biochemical failure [9].

Biochemical failure has been associated with perineural inversion [10], but there are conflicting reports [11] where a significant association has not been demonstrated. Vascular invasion has been reported to be associated with biochemical failure but added minimally to prediction models incorporating established risk factors during short follow up periods [12].

Our data using CPCs differs from the data reported by Morgan et al. [3] using the detection of DTCs in bone marrow aspirates, where the surgical margin was not associated with DTCs nor was pathological stage. There was a trend for increasing CPC detection frequency associated with increasing serum PSA and increasing Gleason score, which again was not seen in the study of DTCs by Morgan et al. [3]. However, in contrast there are published reports that primary CTCs, DTCs and micrometastasis are not associated with the Gleason score before primary therapy and that DTCs and micrometastasis after primary therapy are associated with Gleason score [5]. What may be important is that although increasing Gleason score was associated with an increased frequency of CPCs detected, in patients after radical prostatectomy those with CPCs had an increased risk of biochemical failure independent of Gleason score. This can be explained by that patients with higher Gleason scores have a higher chance of having subpopulations of cancer cells that can disseminate and implant in distant tissues. However, patients with implanted or micrometastatic cells all have a higher risk of biochemical failure and thus are independent of Gleason score.

Our results differ from those using DTCs, which may be explained in part by the use of differing biomarkers. Morgan et al. used anti-Ber4 anti-epithelial antibody, while we used anti-PSA. In higher grade tumors there can be decreased epithelial antigen expression and if as suggested that DTCs are circulating tumor cells, the transition epithelial mesodermal that is suggested to occur during dissemination may account for decreased epithelial antigen expression. The widely accepted concept that all cytokeratin and/or EpCAM positive, CD45 negative cells with a nucleus in cancer patients are circulating tumor cells (CTCs) has imposed a clear bias on the study of CTCs. Mainly the failure to include tumor cells that have reduced or absent cytokeratin and/or EpCAM expression and the failure to identify such cell types limit investigations into additional tumor types. EpCam is expressed in most but not all tumors [13]; there is downregulation with cancer progression and metastasis; cytokeratins are heterogeneously expressed in tumor cells and also may be downregulated during disease progression or in poorly differentiated tumors. During the progression of epithelial to mesenchymal transition both markers are downregulated [14]; EpCAM may be downregulated to allow epithelial cell dissociation from the tumor and cytokeratin downregulated to facilitate cell plasticity and migration [15]. However, Fizazi et al. [16], using anti-BerEP-4 epithelial antigen combined with telomerase activity, detected primary CPCs in 79% of patients with localized cancer, which suggests that the anti-BerEP-4 may be appropriate to detect DTCs.

To date, there are few published studies evaluating the significance of CPCs in prostate cancer patients after radical prostatectomy. Using rt-PCR in 50 patients it was reported that in men with a rising PSA 47% of patients had CPCs detected in comparison with 3% without a rising PSA [17]. In men with biochemical failure after radical prostatectomy the detection of CPCs was associated with a shorter PSA doubling time [18].

There is a clear need to identify the role of secondary CPCs in prostate cancer and also to determine on a biological level what mechanisms enable prostate cancer to recur after many years without detection. Our results indicate that a large proportion of patients with no evidence of disease have CPCs detectable after surgery, and they may reappear after a period of time of being CPC negative. These positive patients have a higher risk of biochemical failure and it suggests that tumor dormancy plays a prominent role in prostate cancer recurrence after definitive therapy. We suggest that men who become CPC positive after prostatectomy radical have dormant micrometastasis that may eventually activate and cause metastasis.

The observations from our study must be taken in the context of a population of 92 patients and although the median followup is only two years there were sufficient biochemical failures to make some general observations. Firstly CPC detection using standard immunocytochemisrty is able to identify a high risk group for biochemical failure before there is a rise in the serum PSA. By using a positive/negative result and not a defined cutoff point of a determined number of cells/mL blood it gives the treating physician a yes/no answer. The time from surgery does not influence the interpretation of the test in men with a serum PSA <0.2 ng/mL. We sought to maximize sensitivity of the test by utilizing a single CPC cutoff. A higher cutoff would have decreased false positives, but the correlation between CPCs and biochemical recurrence presented here supports the single cell definition.

Obtaining blood samples for CPC detection is less invasive than the use of bone marrow specimens and thus could be more frequently repeated during followup.

In summary secondary CPCs are associated with increased biochemical failure in men with a PSA <0.2 ng/mL after radical prostatectomy; the presence of secondary CPCS is independent of the clinicopathological parameters normally used to predict risk of biochemical failure; however the presence of secondary CPCs does not determine if the recurrence is due to local or systemic disease. These results warrant larger studies to confirm the findings.

## Figures and Tables

**Table 1:** Demographic details of the study population.

	Group 1	Group 2	Group 3	Total
No. of patients	28	64	22	114
Initial stage				
1	9	9	0	
2	16	33	11	
3	3	22	11	
Median Gleason score (IQR)	6 (5-6)	6 (5-6)	6 (5-6)	
Time from surgery (years)	3.8 ± 1.6	3.2 ± 2.9	5.6 ± 3.6	

**Table 2:** Secondary CPC detection and association with clinical parameters.

Clinical parameter	CPC (+)	CPC (−)	
Margin (+)	15	7	*P* = 0.003
Margin (−)	23	47	
Capsule (+)	24	17	*P* = 0.003
Capsule (−)	14	37	
Perineural (+)	27	29	*P* = 0.09
Perineural (−)	11	25	
Vascular (+)	15	3	*P* = 0.00005
Vascular (−)	23	51	
Lymphatic (+)	13	4	*P* = 0.001
Lymphatic (−)	25	50	
Gleason 4	1	11 RR 1.00	*P* = 0.015 for trends
Gleason 5 + 6	31	23 RR 7.41	
Gleason 7	8	11 RR 4.00	
Gleason 8 + 9	8	1 RR 44.00	

**Table 3:** Biochemical failure and association with clinical parameters.

Clinical parameter	BF (+)	BF (−)	
Margin (+)	8	14	*P* = 0.97
Margin (−)	22	48	
Capsule (+)	14	24	*P* = 0.53
Capsule (−)	16	38	
Perineural (+)	25	30	*P* = 0.001
Perineural (−)	5	32	
Vascular (+)	10	8	*P* = 0.02
Vascular (−)	20	54	
Lymphatic (+)	8	8	*P* = 0.08
Lymphatic (−)	22	54	
Gleason 4	0	12	*P* = 0.05 for trends
Gleason 5 + 6	18	36	
Gleason 7	8	11	
Gleason 8 + 9	4	5	

**Table:** (a)

	Con biochemical failure	Total	RR
(a) Margin (+) CPC (+)	9	15	16.5
(b) Margin (+) CPC (−)	1	7	1.83
(c) Margin (−) CPC (+)	17	22	37.4
(d) Margin (−) CPC (−)	4	48	1.0

**Table:** (b)

	Con biochemical failure	Total	RR
(a) Capsule (+) CPC (+)	13	22	12.6
(b) Capsule (+) CPC (−)	1	16	0.58
(c) Capsule (−) CPC (+)	12	15	35.0
(d) Capsule (−) CPC (−)	4	39	1.0

**Table:** (c)

	Con biochemical failure	Total	RR
(a) PN (+) CPC (+)	20	25	100.0
(b) PN (+) CPC (−)	5	30	5.0
(c) PN (−) CPC (+)	5	11	20.9
(d) PN (−) CPC (−)	0	26	1.0

**Table:** (d)

	Con biochemical failure	Total	RR
(a) V (+) CPC (+)	10	16	17.0
(b) V (+) CPC (−)	0	2	3.4
(c) V (−) CPC (+)	15	18	51.0
(d) V (−) CPC (−)	5	56	1.0

**Table:** (e)

	Con biochemical failure	Total	RR
(a) L (+) CPC (+)	8	13	15.0
(b) L (+) CPC (−)	0	3	3.1
(c) L (−) CPC (+)	17	25	20.0
(d) L (−) CPC (−)	5	52	1.0

**Table:** (f)

	CPC (+)	BF	CPC (−)	BF
Gleason 4	1	0	11	0
Gleason 5 + 6	31	16	23	2
Gleason 7	8	5	11	3
Gleason 8 + 9	8	4	1	0

Total				

**Table 5:** Kaplan Meyer plot for men without biochemical failure with time after radical prostatectomy.

	0 years	1 year	2 years	3 years	4 years	5 years	6 years	7 years
CPC (+)	100% 38/38	38/38	26/31	24/28	18/24	13/17	9/14	6/7
CPC (−)	100% 54/54	54/54	52/53	44/44	39/40	28/29	13/14	8/9
		*P* = 1.00	*P* = 0.02	*P* = 0.02	*P* = 0.009	*P* = 0.06	*P* = 0.16	*P* = 1.00

**Table 6:** Uncensored Kaplan-Meier of men without biochemical failure comparing CPC (+) versus CPC from time of blood sampling.

	*T* = 0	*T* = 1 year	*T* = 2 years	*T* = 3 years
CPC (+)	100% (38/38)	74% (28/38)	45% (9/20)	20% (1/5)
CPC (−)	100% (54/54)	100% (54/54)	91% (31/33)	77% (14/17)
		*P* = 0.00006	*P* = 0.0001	*P* = 0.02
